# Convergence angles of all-ceramic full crown preparations performed in Dubai private practice

**DOI:** 10.4317/jced.55269

**Published:** 2018-12-01

**Authors:** Fahad Abdulla, Hassan Khamis, Alexander Milosevic, Moosa Abuzayda

**Affiliations:** 1 BDS MSc Former resident, Department of Prosthodontics, Hamdan Bin Mohamed College of Dental Medicine, Mohamed Bin Rashid University (MBRU) of Medicine and Health Sciences, Dubai, UAE; 2 BSc MSc DEA PhD, Department of Biostatistics, Hamdan Bin Mohamed College of Dental Medicine, Mohamed Bin Rashid University (MBRU) of Medicine and Health Sciences, Dubai, UAE; 3 BDS PhD FDSRCS DRDRCS FDTF Ed, Chair and Program Director, Department of Prosthodontics, Hamdan Bin Mohamed College of Dental Medicine, Mohamed Bin Rashid University (MBRU) of Medicine and Health Sciences, Dubai, UAE; 4 DDS Dr Med Dent, Department of Prosthodontics, Hamdan Bin Mohamed College of Dental Medicine, Mohamed Bin Rashid University (MBRU) of Medicine and Health Sciences, Dubai, UAE

## Abstract

**Background:**

This study aimed to determine the degree of taper and total occlusal convergence angles (TOC) for all-ceramic bonded crown preparations carried out by private practitioners in Dubai, UAE.

**Material and Methods:**

A convenience sample of all-ceramic crown preparations carried out by private dental practitioners were scanned (Carestream CS 3500) from casts and the digital images assessed. The degree of taper was measured on the axial walls of each crown preparation and the bucco-lingual and mesio-distal convergence angles subsequently calculated.

**Results:**

A total of 154 dentists prepared a total of 206 crown preparations (72 anterior, 134 posterior). The mean convergence angles mesio-distally for all preparations was 24.6° (sd 11.8º), and for the bucco-lingual it was 32.6° (sd 15.3°). The mean TOC was 28.6°. In anterior preparations, the mean bucco-lingual convergence angle was 38.8° (sd 12.2°) compared to 29.3° (sd 15.5°) for posterior preparations (*p*<0.001). Mean mesio-distal convergence anteriorly was 20.6° (sd 10.18°) compared to 26.7° (sd 12.16°) posteriorly (*p*<0.001). Distal and buccal taper were significantly greater on posterior teeth (<0.001) compared to anteriors whereas lingual taper was greater on anterior teeth (*p*<0.001). Mesial taper was not different. Premolars had significantly lower convergence values compared to other teeth.

**Conclusions:**

Bucco-lingual and mesio-distal convergence angles significantly exceeded the clinically acceptable convergence angle of between 10° and 22°. Greater axial taper is recommended for resin bonded all-ceramic crowns but reliance on adhesion in such preparations rather than parallelism may reduce retention and have increased biologic cost to pulp health.

** Key words:**All-ceramic crown preparations, convergence angles, axial taper.

## Introduction

The retention of a single crown relies on several factors, such as the height of the preparation, surface texture, the method of placement (cemented or bonded), the closeness of fit, and the axial taper of the preparation walls. The total occlusal convergence angle (TOC), however, represents the most fundamental factor contributing to retention of crownwork and is the angle formed at the intersection of tapers between two opposite axial walls in a given plane (1). The degree of taper and the convergence angle are thus inextricably linked. Achieving axial preparation walls that are as parallel as possible will enhance retention but this can be hindered by various factors, including visibility, accessibility, location and anatomy of the tooth (2).

Jorgensen investigated the relationship between retention and axial wall taper, and noted that retention increased as convergence decreased and recommended an ideal convergence of 5° but also advised some degree of axial convergence was necessary to ensure full seating of cast crowns (3). Full coverage cast preparations are recommended to have 10° to 20° of total occlusal convergence with a minimal height of 4mm for molars and 3mm for other teeth (4,5). Further research led to the conclusion that 16° was the optimal convergence angle, because a 22° convergence provided inadequate resistance and a 10° convergence did not significantly increase retention (6-9).

These early publications focused on retention of cemented metal-ceramic crowns but with the introduction of all-ceramic crowns that are bonded rather than cemented, a greater degree of taper has been accepted. Thus a total occlusal convergence angle of 20° was found not to affect internal fit of zirconia copings for all-ceramic crowns (10). Also a 12° ‘preparation angle’, presumably axial taper, for zirconia copings resulted in the best precision of fit compared to 4° or 8° tapers and had no influence on marginal adaptation (11). The use of adhesive luting resin enhanced the retention values by 20% at 24° taper compared to the retentive values of conventional cements at 6° taper (12). Crown retention using three different tapers (5°, 12°, 25°) and 4 types of lute: zinc phosphate cement, glass ionomer cement, or adhesive resin (Panavia 21 and C&B-Metabond) found that the best retention was obtained when complete metal crowns were cemented with adhesive resin cements, regardless of tooth preparation taper (12). Three studies assessed the quality of metal-ceramic crown preparations provided privately in the Middle East and found that the TOC angles were higher than recommended with the highest recorded value being 38.2° for mesio-distal convergence on molars (13-15). There have been no studies conducted on all-ceramic preparations in the UAE.

This study aimed to determine the degree of taper and therefore total occlusal convergence angle on casts of teeth prepared for all-ceramic crowns by private practitioners in Dubai, UAE. Statistical analysis was performed using SPSS v20. Differences in the convergence angle and axial wall taper values between two groups of teeth (anterior vs. posterior; maxillary vs. mandibular) were tested by independent sample t-test with statistical significance set at *p*<0.05.

## Material and Methods

This was a cross-sectional observational study of the convergence angles on die stone casts of full coverage crowns prepared by dental practitioners in private practice with at least five years of post-graduate experience. The dentists did not know when the casts were to be examined and anonymity of patients and dentists was maintained. Local Research Ethics approval was gained (Ref. EC0615-003). The dies of the crown preparations were obtained from the largest two dental laboratories in Dubai. The die models were not randomly selected but were a convenience sample as some dentists declined to participate. The results are thus not representative of all-ceramic crown work carried out in Dubai. All the stone casts were prepared in a standardized manner using type IV die stone. Serial numeric coding was used for the purpose of die identification.

-Scanning Procedure

The prepared dies were first scanned and digitized with an optical intra-oral scanner (Care Stream CS 3600, Carestream Dental, Atlanta, GA 30339, USA). The 3D digitization of each preparation was evaluated for total occlusal convergence angle both mesio-distally and bucco-lingually. Furthermore, the axial wall tapers for each preparation mesially, distally, buccally and lingually were measured. This was facilitated using CS model software from Carestream (www.carestreamdental.com). There is no standardized technique to measure crown taper although a recent systematic review concluded that the TOC was the most important preparation parameter (16).

**Figure 1:**
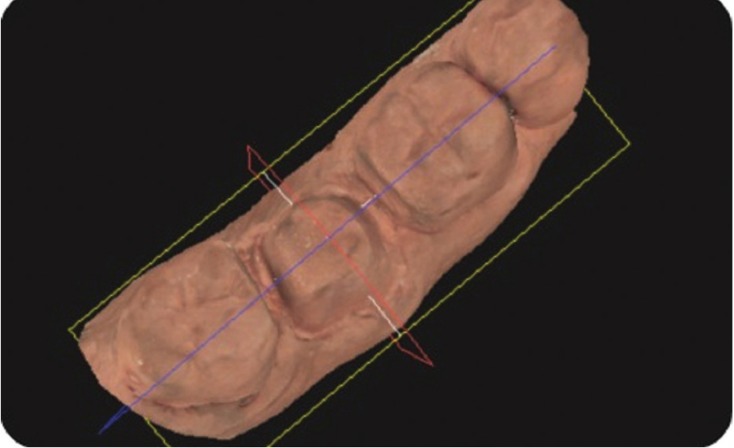
Scanned image of lower second molar showing mid-bucco-lingual plane for subsequent angle measurement on image as shown in next figures.

**Figure 2:**
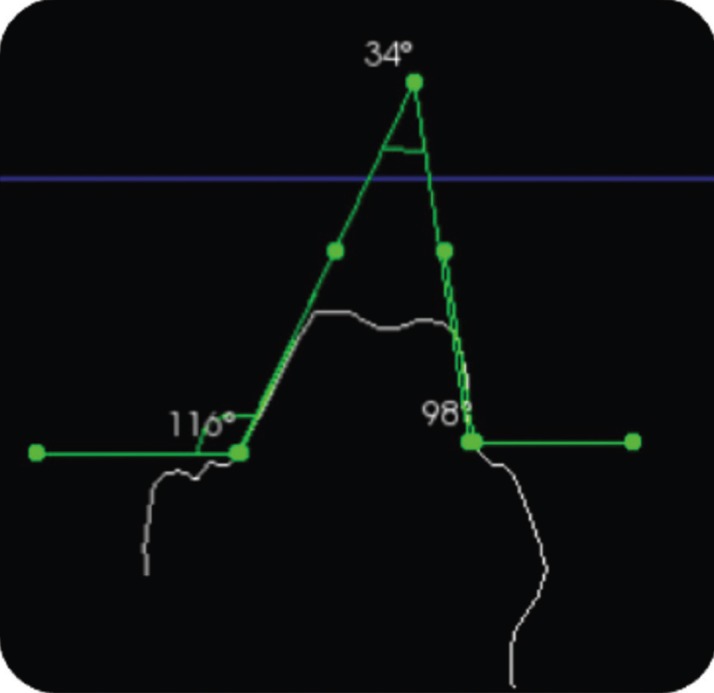
Determination of total occlusal convergence angle on lower molar. Buccal inclination in relation to horizontal plane= 116º. Lingual inclination in relation to horizontal plane= 98º. Total convergence angle bucco-lingually = 34º.

**Figure 3:**
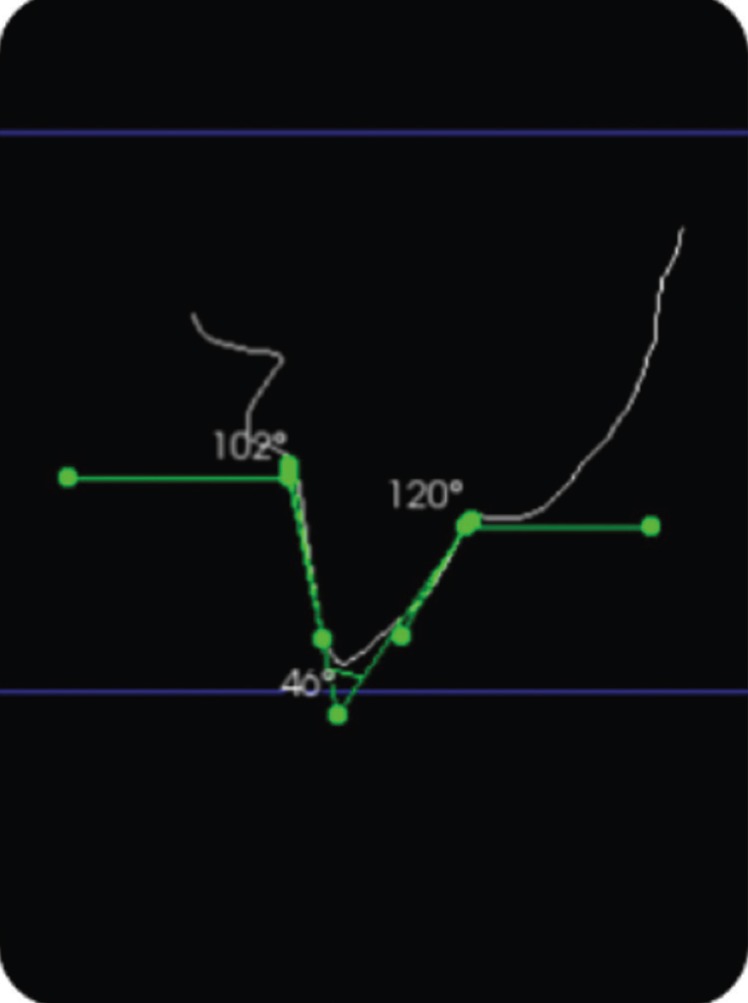
Determination of 46° convergence angle on upper incisor from labial taper at 120° and palatal taper at 102°.

The standardized reference axes were the mid lines on each surface as determined by the software, which made a plane slice through the image perpendicular to an occlusal grid reference (Fig. 1). Bucco-lingual and mesio-distal angles were calculated by measuring the angles formed by drawing straight lines along the axial inclination of the opposing axial surfaces. Individual axial taper, in contrast, was calculated by measuring the angle of axial inclination of each side in relation to the horizontal plane. This was followed by subtracting it from 90º, which represented the angle between the axial inclination and the vertical plane (Figs. 2,3). The formulae to determine the total convergence angle and the axial wall taper are as follows:

1. Total convergence angle = The angle formed when the two lines along the axial wall inclinations meet, either in bucco-lingual or mesio-distal distal cross sections.

2. Axial wall taper = The angle of the axial inclination in relation to the horizontal plane – 90º, which represents the point where the taper inclination started from the vertical plane.

## Results

A total of 135 dentists prepared 135 single crowns, while 19 other dentists prepared 71 multiple preparations. Thus,154 dentists prepared a total of 206 crown preparations. The overall mean total occlusal convergence angle and axial wall taper values were 28.6° ± 10.8° and 14.3° ± 5.4° respectively. The mean mesio-distal and bucco-lingual angles of all preparations were 24.6° and 32.6° respectively and are presented in  Table 1. Mesial, distal, buccal and lingual axial wall tapers are also shown.

**Table 1:**
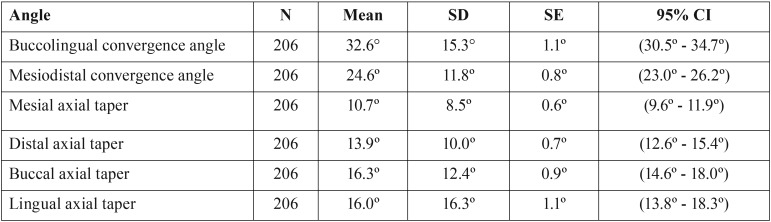
Mean values for convergence angle and axial wall taper angle on all crown preparations.

Anterior teeth had significantly higher mean convergence angles bucco-lingually compared to posterior teeth, whilst posterior teeth had higher mean convergence angles mesio-distally compared to anterior teeth ( Table 2, *p*<0.001). There was no statistical difference in the mean mesial axial taper between anterior and posterior preparations. The distal and buccal axial tapers were significantly higher in posterior teeth compared to the anterior preparations. Conversely, mean buccal or labial wall anterior taper (10.2°) was significantly lower than the equivalent posterior taper value (19.6°) as shown in ( Table 2). In anterior teeth, the lingual/palatal wall taper had the highest degree of taper while buccal, distal and mesial axial wall tapers had similar values. Posterior mean axial wall taper values were 19.6° buccally and 9.4° for the lingual walls.

**Table 2:**
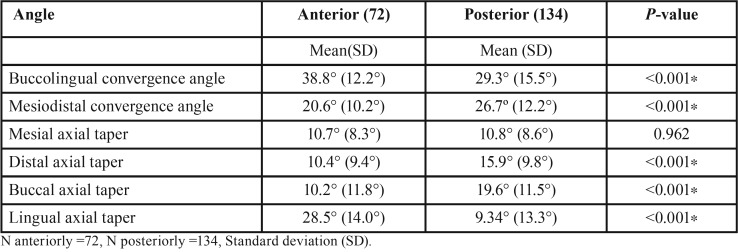
Mean convergence angle and taper values of anterior and posterior teeth.

Mean convergence angle and axial wall taper values differed significantly between maxillary and mandibular teeth as shown in  Table 3 (*p*<0.05). Maxillary teeth had significantly higher bucco-lingual convergence and lingual axial taper values, compared to mandibular teeth. On the other hand, mandibular teeth had significantly higher mesio-distal convergence and distal axial taper values. There was no statistical difference between the two groups in relation to mesial and buccal axial tapers. In the maxillary arch, the lingual taper was the highest compared to the other axial wall inclinations. In the mandibular jaw the distal taper was the highest while mesial taper was the lowest.

**Table 3:**
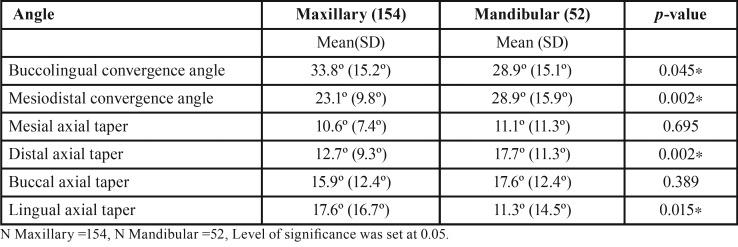
Mean convergence angle according to jaw.

The overall mean convergence and taper angles by tooth type are shown in  Table 4. Bicuspids or premolars had lower angles than those recorded for incisors, canines and molars.

**Table 4:**
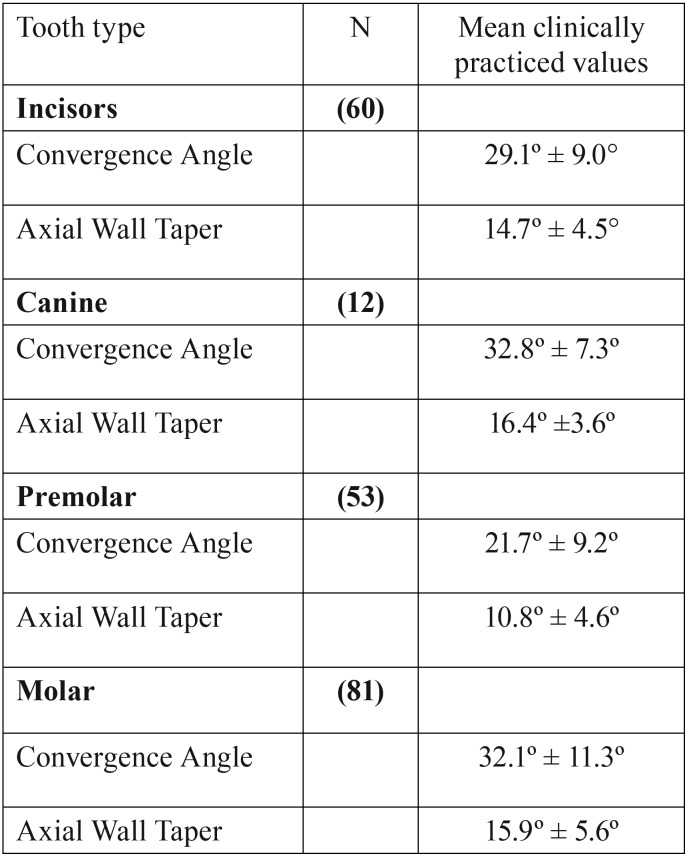
Mean overall convergence and taper values by tooth type.

## Discussion

In this study, the mean of total occlusal convergence angle in 206 preparations was 28.6° against the recommended maximum for all-ceramic crowns of 20°, which although significantly higher, is similar to the results found in previous studies. Clinically, however, adhesive crowns still function adequately even in the presence of high convergence angles. This was explained by the fact that intra-oral forces are more complex than those reproduced in laboratory tests (17,18). In the current study, convergence angles were high and ranged widely according to jaw, tooth type and whether anterior or posterior. These results corroborate previous findings but clinically, since all the preparations were for bonded ceramic crowns, retention may not have been compromised but tooth vitality may have been. Evidence of peri-radicular radiographic change was found in 87 (19%) of 458 vital crown preparations (presumably all were metal-ceramic) mainly on maxillary incisors, maxillary premolars and mandibular molars (19). It is thus noteworthy to link that result with the findings here that incisors and molars had very high convergence angles, which reflect as over-cut preparations that could lead to compromised pulp health. The position of the tooth in either the upper or lower jaw also influenced the convergence angle. In the lower jaw the presence of the tongue may be an obstacle during preparation, compared to the upper teeth. Furthermore, tooth anatomy may have had an impact on convergence angles. Incisors are usually easier to access and thus to prepare, with lower convergence mesio-distally but because of palatal cingula, higher convergence angles labio-palatally are likely in upper anterior teeth. Difficulty angling the hand piece during molar preparation can lead to increased taper, especially distally. Premolars are easier to access than molars, which may account for the more acceptable TOC.

It was not possible to assess differences between vital and non-vital teeth. A microscope assessment of preparations found that the metal-ceramic crown convergence angle for non-vital teeth was greater and ranged between 12° to 37° compared to vital teeth with a range of 19° to 27° (2). The mean angles in the current study lie within the range proposed for non-vital teeth. Several studies have found that the clinically established mean convergence angle among dental students and general practitioners ranged between 12° and 26° and that there is wide variation in convergence angles among general practitioners (20). A mean convergence angle of 24.2° (± 11.95°) on 125 metal-ceramic crowns performed by undergraduate dental students on patients, as opposed to typodonts, was regarded as similar to that produced by experienced dentists (21). Thus experience itself may not be a factor in over-preparation.

The mean convergence values were 32.6° and 24.6° for the bucco-lingual and mesio-distal convergences. This is in agreement with the literature, in which nearly all operators tend to produce greater convergence bucco-lingually. The suggested reasons are: 1) Removal of more buccal tooth structure in order to eliminate molar bulbosity/survey line or undercut and 2) poor palatal cingulum management in anterior maxillary teeth albeit for metal ceramic preparations (22,23). The mesio-distal value was comparable with other studies reporting mean convergence angles practiced by clinicians but the bucco-lingual value exceeded most reported values in the literature (22,24).This could be explained by the fact that previous reports were mainly performed on posterior teeth. The inclusion of 72 anterior tooth in this study has contributed to an increase in bucco-lingual convergence value, since palatal cingula in anterior teeth have influenced the results. This was confirmed with a statistically significant difference between mean anterior bucco-lingual convergence angle (38.8°) and posterior (29.3°). Both of these exceeded the recommended values proposed in earlier studies (4°-14°).

Maxillary teeth had a mean bucco-lingual convergence angle of 33.8° compared to 28.9° for mandibular teeth with a very high palatal mean axial taper of 17.6°. Maxillary bucco-lingual convergence was significantly higher than mandibular teeth possibly because direct vision is more likely for mandibular preparations. Direct line of sight from above and anterior to the long axis of the lower molar is likely to result in an increased distal taper and thus increased mesio-distal convergence.

Premolars (bicuspids) had the lowest convergence values compared to all the other teeth all of which had similar angles. This was contrary to expectations as anterior teeth are the easiest to prepare and were previously reported to have the lowest convergence angles (18). The previous report, however, did not differentiate between maxillary and mandibular incisors and were performed in an academic setting where direct supervision may have aided ideal conservative preparation. The palatal morphology of the upper incisors may predispose to higher convergence angle values. Only 7.8% of all the preparations had a bucco-lingual convergence <12°, while 80% recorded values >22°.

The inter-relationship between preparation height, taper and thermocycling with fracture strength of bonded Lava zirconia crowns was investigated in vitro with the conclusions that taller/less tapered designs were the most retentive and had best fracture strength compared to shorter designs with more or less taper (25). Preparation height is thus an equally important factor when considering crown retention.

Further analysis to determine the effect, if any, of dentists preparing multiple teeth was done as not all data was independent since some dentists (n=19) had prepared more than one tooth (n=71). For this group of dentists, with more than one preparation, results revealed a much greater mean convergence angle value of 38.2° bucco-lingually, but a lower mesio-distal convergence of 20.5°, than the means of all the crowns as shown in  Table 1. This was primarily because of the greater number of maxillary anterior teeth in the sample (58 anterior and 14 posterior teeth) with concomitant very high lingual taper.

## Conclusions

Within the limitations of this study, the mean convergence angles for all-ceramic crowns produced by private general practitioners in Dubai exceeded the recommended guidelines proposed in the literature. The reliance on adhesion at the expense of parallelism in all-ceramic crowns may be detrimental to retention and to pulp health. Further research is needed to determine optimal degree of taper and therefore TOC specifically for bonded crowns as the literature is focused mainly on the long established cemented cast metal-ceramic crown.
